# Heteroresistance is associated with mutations during low concentration of tigecycline therapy in multiple-resistant *Klebsiella pneumoniae*

**DOI:** 10.1186/s12941-025-00815-6

**Published:** 2025-09-30

**Authors:** Qiaoyu Zhang, Linwen Zheng, Lirong Wen, Shanshan Li, Yuli Nie, Jiansen Chen

**Affiliations:** Department of Nosocomial Infection Control, Fujian Medical University Union Hospital, Fuzhou, 350001 Fujian China

**Keywords:** *Klebsiella pneumoniae*, Tigecycline, Resistance, Heteroresistant, Evolution, Time-killing assay, Efflux pumps

## Abstract

**Background:**

Heteroresistance can lead to treatment failure and has brought a rigorous challenge to clinical laboratories for detecting them. The aim of this study was to investigate the potential for tigecycline-susceptible *Klebsiella pneumoniae (K. pneumoniae)* clinical isolates to develop heteroresistance under antibiotic pressure.

**Method:**

In this study, inducing experiment in vitro was used to acquire tigecycline heteroresistance phenotype. Population analysis profiling was used to confirm heteroresistance. Potential tigecycline heteroresistance mechanism through whole-genome sequencing and quantitative reverse-transcription PCR (qRT-PCR) were explored. Time-kill assay was used to explore the effect of tigecycline monotherapy or combination with other antibiotics.

**Result:**

Two clinically isolated *K. pneumonia* strains were found to change from tigecycline susceptible to resistance during treatment of tigecycline in vivo. Experimental-evolved tigecycline heteroresistant colonies were successfully obtained by exposing to concentration of tigecycline at usual therapy of tigecycline (serum concentration of 0.1 mg/L). Heteroresistant phenotypes were stable, and the minimal inhibitory concentration sustained at resistant after 7 days serially passed in tigecycline-free medium. Frequency of heteroresistant subpopulation ranged from 7.0 × 10^−7^ to 1.41 × 10^−6^. Genome sequencing and analysis showed mutations of *ramR*,* acrR* and *rpsJ* could be responsible for the stage from tigecycline susceptible to heteroresistance and further to resistance in *K. pneumoniae*. Quantitative reverse-transcription PCR analysis revealed that the increased expression of tigecycline resistance genes detected in tigecycline resistant subpopulations might be associated with tigecycline heteroresistance. Time-kill assay showed the impaired efficacy of serum concentrations of 0.1 mg/L tigecycline (50 mg/q12h intravenously [i.v.]) monotherapy on tigecycline susceptible *K. pneumoniae*. 1 mg/L tigecycline could be effective in preventing susceptible strain but failed on heteroresistance. Combination with other antibiotics which are susceptible to target strains such as tigecycline-polymyxin B and tigecycline-amikacin can effectively inhibit the growth of resistant subpopulations.

**Conclusion:**

The findings reveal the phenomenon where tigecycline may induce resistance in initially susceptible strains during clinical treatment, associated with several mutations of *ramR*,* acrR and rpsJ*, resulting in treatment failure. The heteroresistant strains induced by low concentrations of tigecycline in vitro provide a perspective for exploring the molecular mechanisms of tigecycline resistance in *K. pneumoniae*. Combination with other antibiotics like polymyxin B and amikacin would show synergistic effects in evading regrowth of resistant subpopulations.

**Supplementary Information:**

The online version contains supplementary material available at 10.1186/s12941-025-00815-6.

## Introduction

*Klebsiella pneumoniae (K. pneumoniae)* is one of the most common pathogens in healthcare facilities, related to the lower respiratory tract, urinary tract and bloodstream infections. This pathogen can easily acquire a variety of resistance mechanisms, and it usually appears as multidrug-resistant (MDR) *K. pneumoniae* in clinical treatment. Tigecycline, as a kind of antibiotic provided with an expanded spectrum of activity against most pathogens, is used to treat MDR *K. pneumoniae* [1]. However, it has been reported that several gram-negative bacteria (GNB), including *K. pneumoniae*, have failed the treatment of tigecycline, and the clinical treatment of such bacterial infections is facing severe challenges.

Tigecycline belongs to a class of antibiotic called glycylcyclines and has a broad spectrum of activity [1, 2]. It is utilized in treating bacteria that produce extended-spectrum β-lactamase or carbapenemase [3]. Similar to tetracyclines, tigecycline reversibly binds to the ribosomal 30 S subunit, blocking translation and inhibiting bacteria growth [4]. It can circumvent the traditional tetracycline resistance mechanisms, including drug efflux mediated by TetA to TetE and ribosomal protection provided by Tet(M) [5]. However, the rising incidence of tigecycline-resistant *K. pneumoniae* is a significant concern. Previous studies have shown that the decreased susceptibility to tigecycline in *Enterobacteriaceae* is due to the overexpression of efflux pumps, especially AcrAB-TolC [6–11]. AcrA, SoxS, and MarA are repressed by RamR, SoxR, and MarR respectively [7, 12]. Since RamR directly suppresses the expression of *ramA*, mutations in *ramR* can result in the overexpression of RamA [7, 13–16]. Furthermore, mutations in the *rpsI and rpsJ* gene, which encodes ribosomal protein S9 and ribosomal protein S10, have been linked to decreased susceptibility to tigecycline [13, 17, 18].

As is considered an intermediate stage during the progression to antibiotic resistance, the occurrence of heteroresistance may lead to the emergence of a resistant strain and ultimately contribute to antibiotic therapy failure. It may be related to the development of antibiotics resistance caused by the sub-inhibitory concentration of tigecycline in vivo, which ultimately leads to the unsuccessful eradication of the pathogen [19–21]. The formation of tigecycline heteroresistance may be related to the selective pressure of irrational use of tigecycline [13]. Here, we report two strains that exhibited heteroresistance under low concentration tigecycline induction. The potential progression of tigecycline heteroresistance mechanism was further explored by in vitro evolution into undertreatment tigecycline.

## Materials and methods

### Strains and antimicrobial susceptibility testing

Tigecycline susceptible *K. pneumoniae* K62, K268 and in vivo-emerged tigecycline resistant *K. pneumoniae* K78, K276 were obtained from Fujian Medical University Union Hospital, a tertiary care teaching hospital with 3500-bed locates in south-east China. Strain K62 and K78 were isolated from urine, strain K268 and K276 were isolated from sputum. *K. pneumoniae* ATCC 13883 was used as a control strain. The strains were stored in *Luria-Bertani* medium (LB, Thermo Fisher Scientific, Shanghai, China) containing with 15% glycerol at −80 °C. In the following-up experiments, the bacteria were re-inoculated on MacConkey Agar (Hopebio, Shandong, China) at 35 °C for 24 h, and a single colony on each agar plate was inoculated into 10 mL of liquid LB medium and incubated at 35 °C for 24 h. The VITEK 2 Compact system (bioMérieux, Marcy l’Etoile, France) was applied for identification of bacterial species and determination of the minimum inhibitory concentration (MIC) of different antibiotics. The breakpoint of tigecycline MICs is based on the criteria established by FDA (≤ 2 mg/L as susceptible, 4 mg/L as intermediate, and ≥ 8 mg/L as resistant).

### Experimental evolution of heteroresistant mutants

The clinical isolates K62 and K268 were cultured on antibiotic-free *Luria-Bertani* agar (LBA, Thermo Fisher Scientific, Shanghai, China) at 35 °C for 24 h. Then isogenic population of each isolate were randomly picked up and resuspended respectively in 5mL *Mueller–Hinton* broth to a concentration of approximately 10^8^ CFU/mL. The bacterial suspension was inoculated into 5mL *Mueller–Hinton* broth (MHB, Oxoid, Basingstoke, United Kingdom) with a 0.1×MIC concentration of tigecycline at the initial inoculum of 5 × 10^5^ CFU/mL. After 24 h exposure at 35 °C, 50µL of culture was isolated and serially diluted from 10^2^ to 10^8^ folds, then spread on MH agar plates. Single clone were randomly picked up and stored in LB medium with 15% glycerol at −80 °C and waiting for the following-up experiments.

### Population analysis profiling and passage stability

Population analysis profiling (PAP) was performed to validate the heteroresistance phenotype as previously described [22]. In brief, 50-µl aliquots of overnight culture were suspended in 5 ml of LB broth and incubated until reaching the mid logarithmic phase (OD600 = 0.3∼0.4). The bacterial pellet was collected through centrifugation and resuspended in MH broth (Oxoid, Basingstoke, United Kingdom). Serial dilutions of the bacterial suspensions were prepared, ranging from 10^8^ to 10^2^ CFU, and spread on MH agar plates (Oxoid, Basingstoke, United Kingdom) containing tigecycline concentration of 0.5, 1, 2, 4, 8, 16, and 32 mg/l. After incubating at 35◦C for 48 h, colonies that emerged on the plates were counted. The frequency of heteroresistant subpopulations was determined by dividing the number of colonies on the highest drug plate by the number of colonies on the corresponding antibiotic-free plate. The experiment was conducted three times, and the mean viable CFU was plotted on a semilogarithmic graph. The detection limit of tigecycline-resistant subpopulations was established at 20 CFU/ml. Tigecycline-heteroresistant *K. pneumoniae* (TGCHR-Kp) was defined as a subpopulation of cells capable of growing at a drug concentration at least twofold higher than those of tigecycline-susceptible parental strains. Subpopulations that grew on the highest tigecycline concentration plates were randomly selected and serially passed daily on antibiotic free medium for 7 days. The MICs were reassessed using the broth microdilution (BMD) method to evaluate the stability of the resistant phenotypes.

### Growth curves and efflux activity inhibition assay

Clinical strain (K62, K268), their evaluated heteroresistant strains (KH62, KH268) and their resistant subpopulations (KH62-R2, KH268-R1) obtained from the PAP were tested. After overnight culture, a 1:100 dilution of saturated culture was added to fresh Mueller–Hinton broth and incubated at 37◦C with shaking for 3 h. Following this, cultures were diluted in cation-adjusted Mueller-Hinton broth (CAMHB) to achieve a turbidity equal to 0.5 McFarland. Subsequently, a 1:100 dilution of each 0.5 McFarland culture was prepared and added to CAMHB, which was continuously shaken at 37◦C for 24 h. The absorbance of the bacterial suspension at 600 nm was measured every hour. Each strain was cultured in triplicate, and the average absorbance values were calculated. The efflux activities were indirectly assessed by agar dilution method with phenylalanine arginine β-naphthylamide (PAβN, Sigma, St. Louis, USA), an efflux pump inhibitor. Briefly, MHA containing a two-fold increase concentration of tigecycline in the presence or absence of 20 mg/L PAβN was prepared. Both the unexposed parental strains and the laboratory evolved tigecycline resistant strains were inoculated on the above plates with 10^4^ CFU per spot. The plates were incubated overnight at 35 °C. If the MIC of tigecycline decreases by 4 times or more after exposure to PAβN, the efflux activity is judged as high. The experiment was repeated three times.

### Real-time RT-PCR analysis and analysis of *ramR*,* soxR*,* marR*,* acrR and rpsJ* mutations

The expression levels of regulator gene *ramA*,* soxS*,* marA* and the efflux components AcrAB-TolC for the strains before and after exposure were assessed by quantitative real-time PCR (qRT-PCR) as previous described [23]. The 2^−ΔΔCT^ method was used to calculate the relative expression of genes by normalizing to the *rrsE* housekeeping gene. Expression of target genes were then calibrated against those of expressed by *K. pneumoniae* ATCC13883.Whole-cell DNA were extracted by boiling method and the *ramR*,* soxR*,* marR*,* acrR*,* rpsI* and *rpsJ* genes were amplified with the primers listed in the previous study [23]. The NCBI BLAST program (http://www.ncbi.nlm.nih.gov/BLAST) was used to align the nucleic acid sequences against the wild-type *K. pneumoniae* reference strain MGH78578 (GenBank accession number CP000647) The sequences had been submitted to GenBank, and the corresponding accession numbers were presented in Table 4.

### Whole-genome sequencing and bioinformatics analysis

We performed the WGS analyses for the heteroresistant parental strain KH62, KH268 and their resistant subpopulation KH62-R2 and KH268-R1 mentioned above. Genomic DNA was extracted, and sequencing libraries were generated. Genome sequencing was then performed by Personal Biotechnology Company (Shanghai, China) by using the Nanopore PromrthION48 platform and the Illumina Novaseq platform. Data assembly was proceeding after adapter contamination removing and data filtering by using AdapterRemoval [24]. The filtered reads were assembled by SPAdes [25] and A5-miseq [26] to constructed scaffolds and contigs. Flye and Unicycler [27]software was used to assemble the data obtained by Nanopore platform sequencing. Finally, the genome sequence was acquired after the rectification by using pilon software [28]. Gene prediction was performed by GeneMarkS v4.32. tRNAscan-SE [29], Barrnap (verison 0.9) and Rfam [30] were used to find tRNA, rRNA and other ncRNA, respectively. CRISPR finders were identified by CRISPR recognition tool. Repeat sequence was analyzed using Repeat Modeler software. For subsystem, PhiSpy [31] and IslandViewer 4 [32]software were respectively used to predict the prophages and genomics islands. Subsequently, the VFDB (Virulence Factors of Pathogenic Bacteria) database, ResFinder 4.7.2 database were used to retrieve the pathogenicity genes and antibiotic resistance genes, respectively (threshold of 90%, minimum length coverage criterion of 60%). The Center for Genomic Epidemiology was used for multilocus sequence typing (MLST), and the sequence types (STs) were determined using the MLST database (https://cge.food.dtu.dk/services/MLST/).The genome sequences of the KH62, KH62-R2, KH268 and KH268-R1 were submitted to the GenBank under accession numbers CP170749, CP171385, CP171444, CP171388.

### Time-kill assay

Antibiotic concentrations used in the time-kill experiments represent the average steady-state concentrations of human non-protein-bound drugs calculated from literature data (based on the area under the antibiotic concentration-time curve in serum or plasma within 24 h divided by 24 h [AUC0–24/24 h]) [33]. The monotherapies were carried out with 0.1 mg/L and 1 mg/L of tigecycline (TGC) [34]; 5.3 mg/L of imipenem (IPM); 8 mg/L and 16 mg/L of amikacin (AMK); 7 mg/L and 10 mg/L of levofloxacin (LEV), 0.25 mg/L and 1 mg/L of polymyxin B(PMB) [35–38]. All antibiotics were purchased from Beijing Solarbio Science & Technology Co., Ltd. (Solarbio, Beijing, China). The time-kill curve was constructed by plotting the amount of CFU grown on the plates. Compared with the initial period, a CFU/mL drop of ≥ 3 log10 at 24 h indicates a bactericidal effect. Bacteriostatic activity is measured by a decrease in colony counts < 3 log10 CFU/mL. Regrowth is defined as an increase in colony counts over the initial point of time [33, 39]. A decrease of > 2 log10 CFU/mL when comparing colonies treated with combined antibiotics and those treated with the most active antibiotic is defined as synergistic effect, while an increase of > 2 log10 CFU/mL considered antagonistic. In this definition, additivity and indifference is defined as any outcome that does not meet the synergism or antagonistic criteria [40]. The detection limit of TGC resistant subpopulations is 20 CFU/mL. All experiments were performed in triplicate.

### Statistical analysis

Statistical analysis was performed using the IBM SPSS 19.0 Statistics software. The statistical analysis of expression of pump genes *acrA*,* acrB*,* tolC*,* oqxB* and regulators *ramA*,* soxS*,* marA* were performed by two-tailed Student’s t-test. A p-value less than 0.05 is considered statistically significant.

## Results

### Characteristics of clinical isolated strains

Emergence of tigecycline resistance after tigecycline treatment in vivo was detected in two patients. The first strain K62 was isolated from the patient’s midstream specimen of urine (MSU) and was susceptible to imipenem (MIC = 1 mg/L) and tigecycline (MIC = 1 mg/L) (Table 1). After intravenous injection of tigecycline (50 mg/q12h) for five days, MSU cultured MDR *K. pneumoniae* (K78), which was resistant to tigecycline (MIC = 16 mg/L). The second isolate (K268) was from sputum, which was only susceptible to tigecycline (MIC = 1 mg/L). After intravenous injection of tigecycline (50 mg/q12h) for nine days, MDR *K. pneumoniae* (K276) were isolated from the sputum, which was resistant to tigecycline (MIC = 16 mg/L) (Table 1). PFGE was performed to identify the homology between the clinical isolates (K62 and K78, K268 and K276). It was shown that K62 and K78, K268 and K276 respectively displayed almost the same PFGE band patterns, revealing the highly homology of the clinical isolates within each patient (Table 1).

**Table 1:** Clinical information and pulsed-field gel electrophoresis pattern (PFGE) for MDR *Klebsiella pneumoniae* strains K62, K78, K268 and K276

PFGE	Isolate	Collect date	Source	Dates of ongoing TGC Treatment(day)	β-Lactamase gene or carbapenamase
	K62	2015-09-17	Urine	0	*bla*_SHV−48_
	K78	2015-09-22	Urine	5	*bla*_SHV−48_
	K268	2016-01-23	Sputum	0	*bla*_KPC−2_
	K276	2016-02-16	Sputum	9	*bla*_KPC−2_

**Fig. 1:**
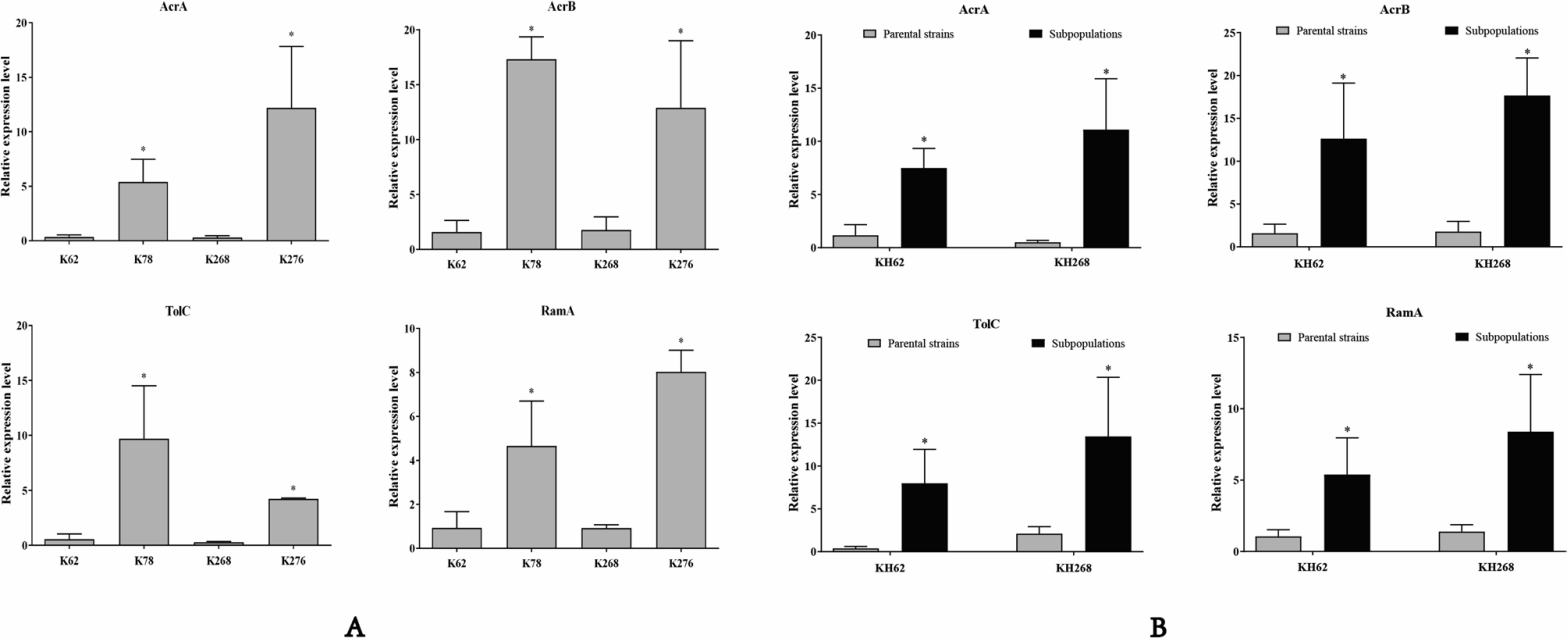
Expression of the efflux pump gene AcrAB-TolC and regulator RamA between clinical isolates, their evaluated TGCHR-Kp and resistant subpopulations. **A** The relative expression of AcrAB-TolC and RamA genes between K62 and K78, K268 and K276 were measured by real-time PCR and normalized to those of *rrsE* housekeeping gene by 2^−ΔΔCT^ method. **B** The relative expression of AcrAB-TolC and RamA genes between evaluated TGCHR-Kp KH62, KH268 and their resistant subpopulations. The expression level is the average value of the three subpopulations. The experiment was repeated three times. Mean relative expression (delta CT values) and standard deviation (SD) are shown. The statistically significant difference is represented by * means *p* < 0.05

Then the expression of pump genes and mutations of *ramR*,* soxR*,* marR*,* acrR*,* rpsI* and *rpsJ* genes between two pairs of clinical isolates were further investigated. The point mutations A19V and F48S in *ramR* were found in K78 and K276, which showed increased expression levels of *ramA* (Fig. 1). Accordingly, compared with tigecycline-susceptible isolates (K62, K268), overexpression of the efflux pump AcrAB-TolC gene (*acrA*, *acrB* and *tolC*) was observed in K78 and K276 (Fig. 1). Neither the efflux pump *oqxB* genes nor the regulatory genes *marA* and *soxS* showed overexpression(Figure S2). Moreover, in the K276, mutations in the *rpsJ* gene and an inserted sequence IS*Kpn26* in the *acrR* gene were also found (Table 4).

### TGCHR-Kps occurrence after in vitro evolution under tigecycline pressure

**Fig. 2:**
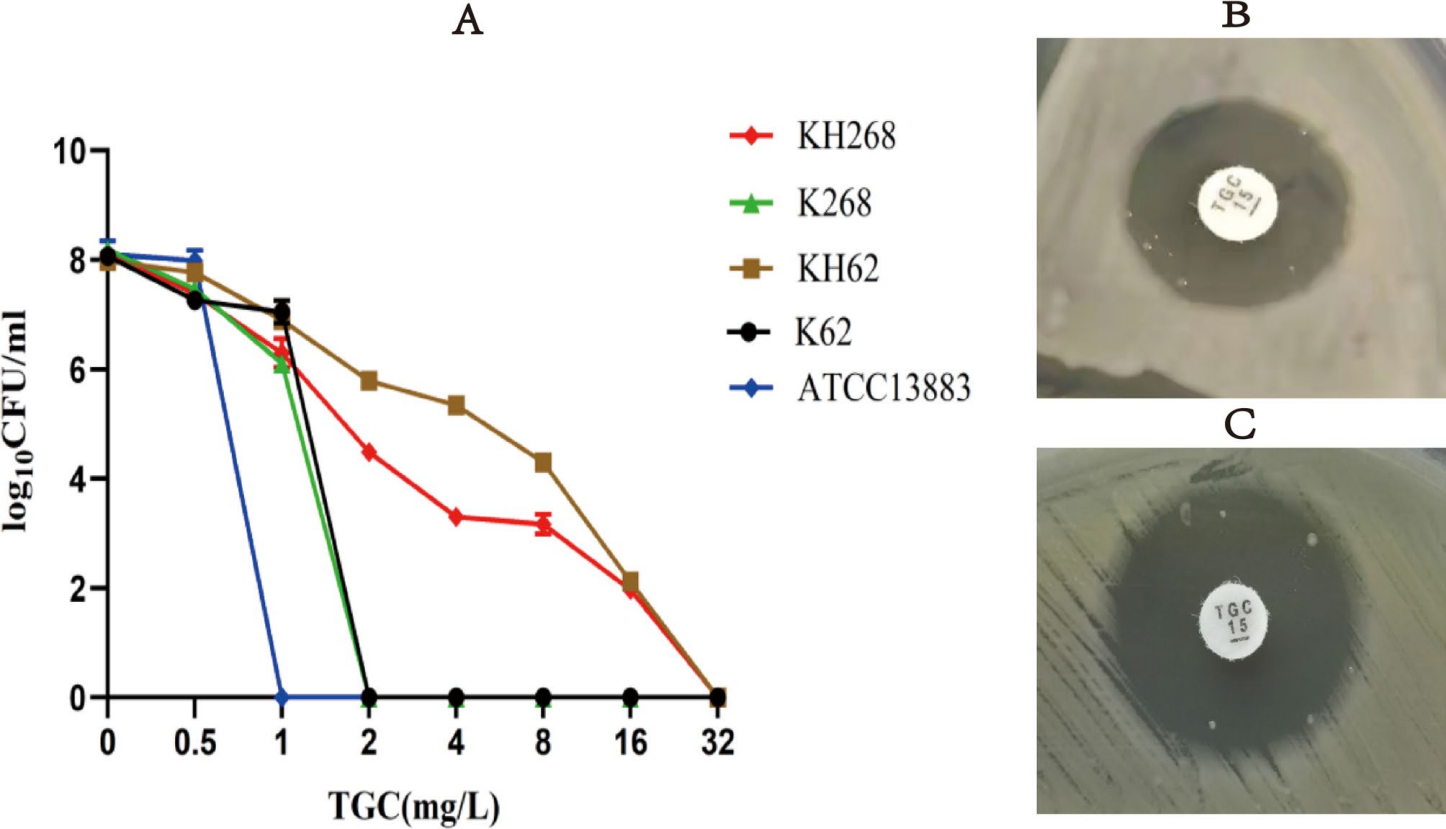
PAP confirmation of evaluated tigecycline heteroresistant *K. pneumoniae* isolates. PAP is described in Materials and Methods. ATCC13883 was used as the control strain. **A** PAP was performed in triplicate to verify TGCHR-Kps in two clinical isolates and their evaluated strains; **B** and **C** The colonies were dispersed within the inhibition zone created by the TGC-containing disk for clinical isolate KH62 (**B**) and KH268 (**C**)

After exposure by serum concentrations of 0.1 mg/L tigecycline(50 mg/q12h) in the laboratory for 24 h, isolates were sub-cultured in LB plate without antibiotic. 8 independent colonies were randomly selected from K62 and K268 respectively and heterosistance of tigecycline was assayed by PAP method. Only one colony of K62 and K268 (KH62 and KH268) performed phenotypic heteroresistance to tigecycline, the resistant subpopulations of the tested heteroresistant strains could grow in tigecycline concentrations as high as 16 mg/L (Fig. 2). The subpopulations grown in the highest tigecycline concentration reached 16-fold MIC increment (16 mg/L) compared to their parental strains, respectively. The parental strain KH62 and subclone KH62-R2 belonged to ST2028. KH268 and KH268-R1 belonged to ST11. The proportion of resistant subpopulations ranged from 7.0 × 10^−7^ to 1.41 × 10^−6^ (Table 3). Moreover, the addition of PAβN reduced the MIC of tigecycline from 8-fold to 32-fold in all the subpopulations, indicating the efflux pump of survived strains can increase the MIC of tigecycline (Table 3). The antibiotic MICs of every TGCHR-Kp and its subpopulation are presented in Table 2. To further confirm the role of the efflux pumps in tigecycline-resistant subpopulations, RT-PCR was performed to detect the expression of efflux pumps and their regulator genes. Compared with the parental strains, the AcrAB-TolC efflux pump genes (*acrA*,* acrB* and *tolC*) in the subpopulations were all up-regulated. Overexpression of regulator *ramA* was observed in all tigecycline-resistant subpopulations (Fig. 1).

**Table 2:** Minimum inhibitory concentration (MICs) of various antibiotics against MDR *Klebsiella pneumoniae* strains

Isolate	MIC									
	TGC	IPM	PMB	CTX	FOX	AMK	LEV	SXT	ATM	FOS
K62	1	1	1	256	64	2	1	20	2	256
K78	16	1	1	256	64	2	4	20	16	256
KH62	1	1	1	256	64	2	1	20	2	256
KH62-R1	8	1	1	256	64	2	4	20	16	256
KH62-R2	16	1	1	256	64	2	4	20	16	256
KH62-R3	16	1	1	256	64	2	4	20	16	256
K268	1	16	1	256	64	64	8	320	64	256
K276	16	16	1	256	64	64	8	320	64	256
KH268	1	16	1	256	64	64	8	320	64	256
KH268-R1	16	16	1	256	64	64	8	320	64	256
KH268-R2	8	16	1	256	64	64	8	320	64	256
KH268-R3	16	16	1	256	64	64	8	320	64	256

*TGC* tigecycline, *IMP* imipenem, *PMB* polymyxin B, *CTX* cefotaxime sodium, *FOX* cefoxitin, *AMK* amikacin, *LEV* Levofloxacin, *SXT* trimethoprim/sulfamethoxazole, *ATM* aztreonam, *FOS* fosfomycin

To assess the stability of the tigecycline-unsusceptible subpopulations, they were serially passaged in tigecycline-free medium for 7 days, followed by an evaluation of tigecycline susceptibility. After subculturing onto tigecycline-free medium, six subpopulations either maintained their original MIC values or exhibited a twofold reduction in tigecycline MICs. All the 6 subpopulations’ MIC retained the unsusceptible range (Table 3). To determine the difference in fitness costs between the susceptible strains and resistant subpopulations, we determined the growth curves of the clinical isolates (K62 and K268), evaluated TGCHR-Kps (KH62 and KH268) and their resistant subpopulations (KH62-R2 and KH268-R1). As shown in Figure S1, the growth rate showed no significant differences between the susceptible clinical isolates, parental strain and resistant subclones, suggesting that the resistant phenotype did not impact the fitness of the bacteria.

**Table 3:** Characterization of the TGCHR-Kps phenotypes

Strains	Parental strain MIC (mg/L)	Highest concentration of growth in PAP (mg/L)	Frequency of resistant subpopulations	Resistant subpopulations (mg/L)			Resistant subpopulations MIC after 7-day passages (mg/L)
				Strain	TGC	TGC + PaβN	
KH62	1	16	1.41 × 10^−6^	KH62-R1	8	1	8
				KH62-R2	16	0.5	16
				KH62-R3	16	0.5	8
KH268	1	16	7 × 10^−7^	KH268-R1	16	0.5	16
				KH268-R2	8	0.5	8
				KH268-R3	16	0.5	16

The tigecycline susceptible strains were inoculated on MH agar plates with 2-fold increasing concentration of tigecycline and cultured at 37°C for 48 h.Three strains of each survived on the highest concentration were randomly picked up, then the tigecycline MIC were measured by agar dilution method with or without PAβN to assess the efflux activities.

### Mutations were found in *ramR*,* acrR and rpsJ* genes in the tigecycline-resistant subpopulations

In 2 evaluated TGCHR-Kp strains and their subpopulations, mutations in *ramR*,* soxR*,* marR*,* acrR*,* rpsI* and *rpsJ* relative to the reference sequence of *K. pneumoniae* MH78578 (accession number KY465996) were detected (Table 4). In this study, we found several kinds of mutations of *ramR* gene among the subpopulations, while all of parental strains presented wild-type *ramR* sequence. The amino acid substitution of *ramR* was detected in subpopulations of KH62-R1 and KH62-R3. Furthermore, a 15 amino acid residues insertion was found in subpopulation of KH62-R2. For the three isogenic subpopulations (KH268-R1 to KH268-R3), the *ramR* mutations were also different from each other. An insertion sequence InsAB and its upstream transposase were inserted into the *ramR* gene of KH268-R1, whereas a different insertion sequence *ISKpn18* was found in KH268-R2. For KH268-R3, except for one amino acid exchange (L13S), no insertion sequence was found. In addition, an insertion sequence IS*Kpn26* was found in the *acrR* gene of subpopulations KH268-R2 and KH268-R3. *rpsJ* mutation (G11ins) was also harbored in KH62-R1 and KH62-R3.

**Table 4:** Mutations in AcrAB-TolC regulators *ramR*, *soxR*, *marR*, *acrR*, *rpsJ* and *rpsI* protein possibly involved in tigecycline-heteroresistant phenotypes(came from Sanger sequencing)

Isolates	ramR	soxR	marR	acrR	rpsJ	rpsI
K62	WT	WT	WT	WT	WT	WT
K78	p. A19V(OK030831)	WT	WT	WT	WT	WT
KH62	WT	WT	WT	WT	WT	WT
KH62-R1	p. A19V(OK030832)	WT	WT	WT	g.G11ins (OK030834)^a^	WT
KH62-R2	p:45 Ins TAAFAQSGIAASTSA (MF324923)^b^	WT	WT	WT	WT	WT
KH62-R3	p. A19V(OK030832)	WT	WT	WT	g.G11ins (OK030833)	WT
K268	WT	WT	WT	WT	WT	WT
K276	p. F48S (OK030830)	WT	WT	p:94 Ins IS*Kpn26* (OK030836)^c^	g. G11ins g. G306ins (OK030835)^d^	WT
KH268	WT	WT	WT	WT	WT	WT
KH268-R1	p:147 Ins transposase (AOZ87176.1) and InsAB (AOZ87177.1) (MF324924)^e^	WT	WT	WT	WT	WT
KH268-R2	p: 90 Ins IS*Kpn18* (MF324925)	WT	WT	p:94 Ins IS*Kpn26* (OK030828)	WT	WT
KH268-R3	p: L13S(MF324926)	WT	WT	p:94 Ins IS*Kpn26* (OK030829)	WT	WT

*WT* wild type, *Ins* insertion, *Del* deletion

a. Insertion of one guanine at position 11 in gene sequence

b. Insertion of 15 amino acids TAAFAQSGIAASTSA at position 45 in amino acid sequence

c. Insertion of element ISKpn26 family transposase at position 94 in amino acid sequence

d. Insertion of one guanine at position 11 and 306 in gene sequence

e. Insertion of connected element InsAB and upstream transposase at position 147 in amino acid sequence

### Whole-genome sequences, general genomic features and nucleotide variants

**Fig. 3:**
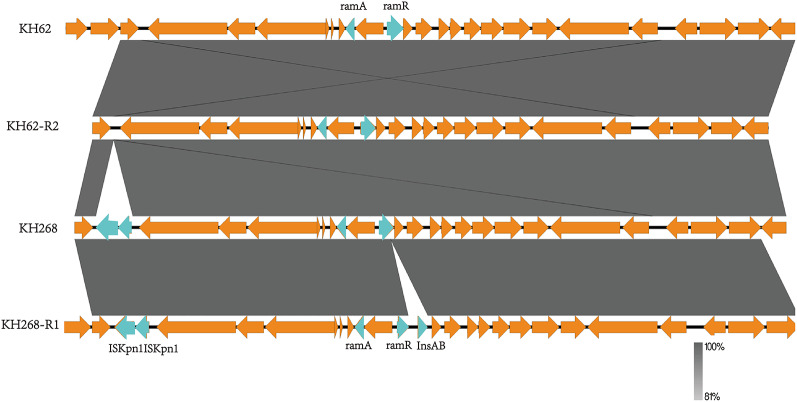
Comparison between the environment of AcrAB-tolC regulators *ramA* and *ramR* genomes of TGCHR-Kp KH62 (top), tigecycline resistant subpopulation KH62-R2 (middle), TGCHR-Kp KH268 (the third) and tigecycline resistant subpopulation (bottom). A zoomed-in view showing that insertion sequence InsAB has been inserted and disrupted the structure of *ramR* in KH268-R1, but not in KH268, nor in KH62 and KH62-R2. Genes and CDs are shown as arrows, and the direction of transcription is indicated by arrowheads. Shared regions with 99% identity are shaded. Regulators and transposase (ISs) genes are shown in red and blue, respectively

In this study, the complete genomic sequences of KH62, KH62-R2, KH268, KH268-R1 were determined. The general genomic features are summarized in Supplementary Table S2-S3 in the supplementary material. A lot of mobile genetic elements were found in KH62, KH268 and their resistant subpopulation, which could transfer horizontally under antibiotic pressure. To investigate the mutations related to tigecycline resistance, pair-wise genetic relatedness among resistant subpopulations and their corresponding parental strains was analyzed using Mauve. For KH62 and KH62-R2, 24 gaps and 26 snp were found. However, no alteration of mutants occurred in genetic loci encoding for proteins with a variety of important functions except a 15 amino acid residues insertion was found in *ramR* of KH62-R2 and inactivated the *ramR*. For KH268 and KH268-R1, 7 gaps and 12 snp were found. No alteration of mutants occurred in genetic loci encoding for proteins with a variety of important functions except an insertion sequence InsAB and its upstream transposase was identified in the genome of KH268-R1 that disrupted the *ramR* (Fig. 3). These results were consistent with the results of the sanger sequence. Furthermore, we could not identify other amino acid alterations capable of conferring tigecycline resistance, such as in the tigecycline binding sites in the 16 S rRNA and ribosomal protein S9 or S10; global regulators AcrR, MarA, and SoxA; or the outer membrane proteins OmpK35 and OmpK36.

### In vitro activity of tigecycline monotherapy or combination with imipenem, levofloxacin, amikacin and polymyxin B

**Fig. 4:**
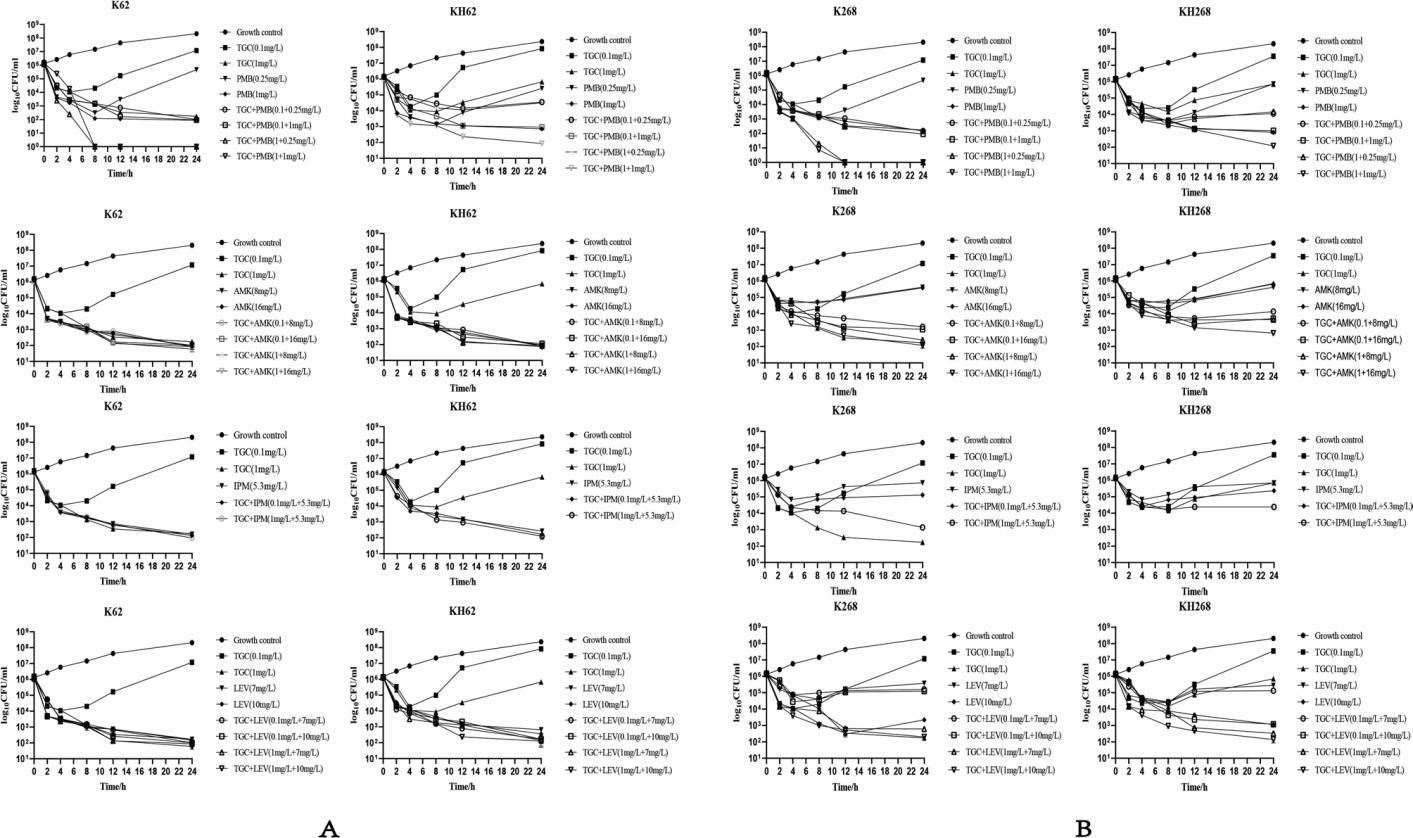
Time-kill curves of different combination therapies against strains with (KH62 and KH268) or without (K62 and K268) tigecycline heteroresistance. In vitro time-kill assays using serum concentrations of polymyxin B (PMB), amikacin (AMK), imipenem (IPM) and levofloxacin (LEV), either alone or in TGC combination against 2 clinical MDR isolates. **A** Monotherapies and combination therapies against the K62 and KH62 isolate, respectively; **B** Monotherapies and combination therapies against the K268 and KH268 isolate, respectively

In strains K62 and K268, TGC (0.1 mg/L) or PMB(0.25 mg/L) monotherapy only reduced ≤ 3 log10 in the initial 6 h, and re-growth significantly upon 24 h, while 1 mg/L TGC or 1 mg/L PMB monotherapy could reduce ≥ 3 log10 and maintain effectiveness for up to 24 h. Combination of low concentration of TGC(0.1 mg/L) and PMB(0.25 mg/L) showed synergistic bactericidal activity within the initial 8 h, and re-growth was not observed upon 24 h. AMK monotherapy had bactericidal effect on K62 at both 8 mg/L and 16 mg/L, but failed on K268, which MIC of AMK was 64 mg/L. While synergistic effect was showed in K268 with TGC-AMK combination even at low concentration of TGC(0.1 mg/L) (Fig. 4). Also, we could find synergistic effects on TGC-LEV combination in K268, while LEV does not exhibit effective antibacterial activity using alone. In TGCHR strains KH62 and KH268, 0.1 mg/L and 1 mg/L TGC reduced ≤ 3 log10 in the initial 6 h but re-growth significantly upon 24 h, combination of TGC(1 mg/L) with other sensitive antibiotics can effectively prevent regrowth.

## Discussion

Previous research has suggested that under tigecycline pressure, bacteria are easily induced to develop tigecycline resistance [13]. In this study, we obtained two pairs of MDR *K.pneumoniae* strains that were isolated from two clinical patients before and after tigecycline treatment. With treatment by commonly used of tigecycline (50 mg/q12h intravenously [i.v.]) for 6 and 9 days, the tigecycline MICs respectively increased from 1 mg/L (K62 and K268) to 16 mg/L (K78 and K276). Overexpression of RND efflux pumps AcrAB-TolC/OqxAB and mutations in the ribosomal protein (*rpsI* and *rpsJ*) have been reported as the most important mechanisms that result in tigecycline resistance in *K. pneumoniae* [7, 13, 14]. In our study, reduction of MIC values from 16 mg/L to 1 mg/L was obtained for tigecycline resistant strains K78 and K276 collected from clinical patients after using the efflux pump inhibitor. Furthermore, overexpression of RND efflux pumps AcrAB-TolC has been found in K78 and K276, suggesting that tigecycline resistance is related to the upregulated AcrAB-TolC efflux systems. The AcrAB-TolC efflux pump systems in the *Enterobacteriaceae* family are regulated by the local regulators AcrR and the global regulators MarA, SoxS, and RamA [6, 41, 42]. The transcription of *marA* is repressed by MarR. As RamR directly represses the expression of *ramA*, mutations in *ramR* can cause the overexpression of RamA. Similarly, *soxS* induction depends on SoxR, and its overexpression can be led by mutations within *soxR* [41]. In our study, a substitution mutation A19V was found in *ramR* of tigecycline resistant isolates K78 (Table 4), resulting in overexpression of *ramA* compared with K62 with wild type *ramR* (Fig. 1). The A19V *ramR* substitution mutation was dominant (4/23, 17%) in a study in China [43], it was also reported in other studies [44]. A substitution mutation F48S of *ramR* was also found in another tigecycline resistant isolate K276. Moreover, an insertion element IS*Kpn26* integrated into the *acrR* gene of K276. IS*Kpn* elements are important mobile mediators to rewiring genomic expression and causing gene inactivation [45]. IS*Kpn26* has been commonly found in *K. pneumoniae* and played a significant role in genome evolution [45, 46]. Yang et al. reported a strong link between KPC-2 plasmid-located IS*Kpn26* and IS*Kpn26* insertion into *acrR*, indicates that *bla*_KPC−2_ plasmid is the reservoir for IS*Kpn26* [47]. Mutations of the ribosomal S9 and S10 protein gene *rpsI* and *rpsJ* are also a generate target that has been described to decrease tigecycline susceptibility in Gram-negative bacteria [17, 18]. Our study found an insertion position occurs at point 305 in *rpsJ* of K276, which may play a role in tigecycline resistance. However, no mutations of *rpsI* were found in K78 and K276.

As an intermediate stage in the complete transition from susceptibility to resistance, heteroresistance could evade laboratory testing, leading to treatment failure [48]. Tigecycline resistance among *K. pneumoniae* has been reported. However, the study of tigecycline heteroresistance in *K. pneumoniae* is limited. In our previous studies, we found that among 268 clinical tigecycline susceptible *K. pneumoniae* strains, 21 were confirmed heteroresistant to tigecycline [23]. Furthermore, 66.2% of clinical carbapenem-resistant *K. pneumoniae* isolates exhibited TGC-heteroresistant phenotypes [49]. Randomized trials have indicated that compared to bactericidal drugs, bacteriostatic antibiotics are more likely to promote the emergence of heteroresistance and confer an increased mortality risk [50]. Until now, fewer studies successfully evolved tigecycline resistant survivors from clinical susceptible *K. pneumoniae in vitro* [13]. However, the induction of heteroresistant to tigecycline still remains further research.

Here we successfully obtain tigecycline heteroresistant parental isolates (KH62 and KH268), by exposing two clinical susceptible isolates K62 and K268 to serum concentration of tigecycline(0.1 mg/L) cultures *in* vitro, suggesting that monotherapy of tigecycline with low concentration could cause adaptive resistance in *K. pneumoniae in vitro.* The frequency of the resistant subpopulation occurring in KH62 and KH268 is very low, suggesting that conventional laboratory testing may have difficulty detecting it (Table 3). All six selected tigecycline subpopulations of KH62 and KH268 had an up to 32 folds decrease for tigecycline MIC in the presence of PAβN (Table 3), indicated the important role of overexpression of efflux pumps in decreased tigecycline susceptibility, as the same with clinical isolated tigecycline resistant strains K78 and K276. Varies mutations in *ramR* occurred in all subpopulations, upregulated expression of *ramA* and efflux pump AcrAB-TolC, further demonstrated the attribution of overexpression of efflux pump. Noteworthy, for three isogenic colonies selected from clinical strain K268(K268-R1 to K268-R3), three different insert sequences (InsAB, IS*Kpn18* and IS*Kpn26*) were found to disrupt gene structures of *ramR* and *acrR*. This suggests that there is more than one insertion sequence among KH268, and the horizontal transfer of these elements may be involved in the emergence of bacterial heteroreistant strain. The whole genome sequencing results also confirmed the presence of several insertion sequences in KH268, some of which are located near the *ramA-ramR* regulatory elements, further increasing the likelihood of heteroresistance to TGC (Fig. 3 and Table S3).

AcrAB-TolC system is a multidrug efflux pump and can export a variety of antibiotics, such as β-lactams, sulfonamides, and tetracyclines, reducing the effectiveness of these antibiotics and leading to bacterial resistance. Our study showed that due to the overexpression of AcrAB-TolC, the MIC of LEV and ATM for the multidrug-resistant strain K78 or heteroresistant subpopulations were both increased (Table 2). However, the AMK MIC did not increase. Previous studies have shown that AcrAB-TolC is primarily associated with tetracycline resistance but not the major mechanism associated with aminoglycoside resistance. As a member of the RND family, the AcrAD-TolC system plays a major role in aminoglycoside antibiotics.

As a kind of bacteriostatic agent, TGC always be considered that can simply arrest bacteria’s growth rather than killing [51], although it is recognized that at elevated concentration, bacteriostatic antibiotics have the ability to kill bacteria. Selective pressure on bacteria upon using bacteriostatic antimicrobials alone could lead to the development of antimicrobial resistance because of emerging heteroresistance. In our research, the bacteriostatic effect on K62 and K268 upon using TGC alone was found when concentration was 1 mg/L. However, TGC-heteroresistant isolates KH62 and KH268 showed a ≤ 3 log10 reduction at the first 8 to 10 h of TGC mono-treatment and then significantly resumed growth after an extended incubation until 24 h. This result was consistent with previous studies that heteroresistance may contribute to the failure of TGC treatment [52, 53]. PMB has been widely used as the last treatment resort against the spread of superbugs [54, 55], our findings confirmed that the addition of PMB at low concentration(0.25 mg/L) could enhance the antimicrobial effect of TGC, especially against strains heteroresistant to TGC (Fig. 4). This result was consistent with previous studies [49]. Previous in vitro and in vivo studies showed TGC-AMK combination could effectively suppress the selection of resistance at low concentrations [56–58]. For TGCHR KH62 and KH268, synergism effects occurred when 0.1 mg/L or 1 mg/L TGC combined with AMK. 1 mg/L TGC combined with LEV also has a synergistic effect, but 0.1 mg/L is ineffective. It is also notable that the combination between TGC with IPM showed no significant antibacterial effect. Given that combination of 1 mg/L TGC with antibiotics PMB, AMK or LEV can effectively prevent regrowth of resistant TGC subpopulations, when the target bacteria are susceptible to these antibiotics, it is reasonable to explore combination therapies of TGC with these antibiotics to prevent the emergence of mutations and resistant subpopulations based on the different MIC of parental strains.

## Conclusion

Based on our study’s conclusions, we suggest bacteriostatic antibiotic tigecycline used alone in treating MDR-Kp would cause the emergence of tigecycline heteroresistant isolates, associated with several mutations of *ramR*,* acrR and rpsJ.* Combination with other antibiotics like PMB and AMK would show synergistic effects in evading regrowth. Further study is mandatory to assess these regulators to determine their possible regulatory roles in the microbial response to antimicrobial challenges. Using antibiotics reasonably is urgently needed in reducing tigecycline resistance in MDR-Kp.

## Supplementary Information

Supplementary material 1.

## Data Availability

No datasets were generated or analysed during the current study.

## Abbreviations

PAP: Population analysis profiling
WGS: Whole-genome sequencing
qRT-PCR: Quantitative reverse-transcription PCR
MIC: Minimal inhibitory concentration
GNB: Gram-negative bacteria
MDR: Multidrug-resistant
TGCHR-Kp: Tigecycline-heteroresistant *K.* *pneumoniae*
CAMHB: Cation-adjusted Mueller-Hinton broth
BMD: Broth microdilution
PaβN: Phenylalanine arginine β-naphthylamide
TGC: Tigecycline
IPM: Imipenem
AMK: Amikacin
PMB: Polymyxin B
LEV: Levofloxacin
CTX: Cefotaxime sodium
FOX: Cefoxitin
SXT: Trimethoprim/sulfamethoxazole
ATM: Aztreonam

## References

[1] Livermore DM. Tigecycline: what is it, and where should it be used? J Antimicrob Chemother. 2005;56(4):611–4. 16120626 10.1093/jac/dki291

[2] Ahn C, Yoon SS, Yong TS, Jeong SH, Lee K. The resistance mechanism and clonal distribution of Tigecycline-nonsusceptible *Klebsiella pneumoniae* isolates in Korea. Yonsei Med J. 2016;57(3):641–6. 26996563 10.3349/ymj.2016.57.3.641 PMC4800353

[3] Chen YH, Lu PL, Huang CH, Liao CH, Lu CT, Chuang YC, Tsao SM, Chen YS, Liu YC, Chen WY, et al. Trends in the susceptibility of clinically important resistant bacteria to tigecycline: results from the Tigecycline *in vitro* surveillance in Taiwan study, 2006 to 2010. Antimicrob Agents Chemother. 2012;56(3):1452–7. 22203598 10.1128/AAC.06053-11 PMC3294947

[4] Olson MW, Ruzin A, Feyfant E, Rush TS, O’Connell J, Bradford PA. Functional, biophysical, and structural bases for antibacterial activity of Tigecycline. Antimicrob Agents Chemother. 2006;50(6):2156–66. 16723578 10.1128/AAC.01499-05 PMC1479133

[5] Fluit AC, Florijn A, Verhoef J, Milatovic D. Presence of tetracycline resistance determinants and susceptibility to tigecycline and minocycline. Antimicrob Agents Chemother. 2005;49(4):1636–8. 15793159 10.1128/AAC.49.4.1636-1638.2005 PMC1068614

[6] Roy S, Datta S, Viswanathan R, Singh AK, Basu S. Tigecycline susceptibility in *Klebsiella pneumoniae* and *Escherichia coli* causing neonatal septicaemia (2007-10) and role of an efflux pump in tigecycline non-susceptibility. J Antimicrob Chemother. 2013;68(5):1036–42. 23335112 10.1093/jac/dks535

[7] He F, Fu Y, Chen Q, Ruan Z, Hua X, Zhou H, Yu Y. Tigecycline susceptibility and the role of efflux pumps in tigecycline resistance in KPC-producing *Klebsiella pneumoniae*. PLoS One. 2015;10(3):e0119064. 25734903 10.1371/journal.pone.0119064 PMC4348519

[8] Horiyama T, Nikaido E, Yamaguchi A, Nishino K. Roles of *Salmonella* multidrug efflux pumps in Tigecycline resistance. J Antimicrob Chemother. 2011;66(1):105–10. 21081542 10.1093/jac/dkq421

[9] Li J, Zhang H, Ning J, Sajid A, Cheng G, Yuan Z, Hao H. The nature and epidemiology of oqxab, a multidrug efflux pump. Antimicrob Resist Infect Control. 2019;8:44. 30834112 10.1186/s13756-019-0489-3 PMC6387526

[10] Veleba M, De Majumdar S, Hornsey M, Woodford N, Schneiders T. Genetic characterization of Tigecycline resistance in clinical isolates of *Enterobacter cloacae* and *Enterobacter aerogenes*. J Antimicrob Chemother. 2013;68(5):1011–8. 23349441 10.1093/jac/dks530

[11] Veleba M, Schneiders T. Tigecycline resistance can occur independently of the RamA gene in *Klebsiella pneumoniae*. Antimicrob Agents Chemother. 2012;56(8):4466–7. 22644034 10.1128/AAC.06224-11 PMC3421586

[12] Bialek-Davenet S, Marcon E, Leflon-Guibout V, Lavigne JP, Bert F, Moreau R, Nicolas-Chanoine MH. In vitro selection of RamR and SoxR mutants overexpressing efflux systems by fluoroquinolones as well as cefoxitin in *Klebsiella pneumoniae*. Antimicrob Agents Chemother. 2011;55(6):2795–802. 21464248 10.1128/AAC.00156-11 PMC3101381

[13] Fang L, Chen Q, Shi K, Li X, Shi Q, He F, Zhou J, Yu Y, Hua X. Step-wise increase in Tigecycline resistance in *Klebsiella pneumoniae* associated with mutations in ramr, Lon and RpsJ. PLoS One. 2016;11(10):e0165019. 27764207 10.1371/journal.pone.0165019 PMC5072711

[14] Chiu SK, Huang LY, Chen H, Tsai YK, Liou CH, Lin JC, Siu LK, Chang FY, Yeh KM. Roles of RamR and tet(A) mutations in conferring tigecycline resistance in Carbapenem-Resistant *Klebsiella pneumoniae* clinical isolates. Antimicrob Agents Chemother. 2017. 10.1128/AAC.00391-17. 28533243 10.1128/AAC.00391-17 PMC5527587

[15] Hentschke M, Wolters M, Sobottka I, Rohde H, Aepfelbacher M. RamR mutations in clinical isolates of *Klebsiella pneumoniae* with reduced susceptibility to tigecycline. Antimicrob Agents Chemother. 2010;54(6):2720–3. 20350947 10.1128/AAC.00085-10 PMC2876394

[16] Wang X, Chen H, Zhang Y, Wang Q, Zhao C, Li H, He W, Zhang F, Wang Z, Li S, et al. Genetic characterisation of clinical *Klebsiella pneumoniae* isolates with reduced susceptibility to tigecycline: role of the global regulator RamA and its local repressor RamR. Int J Antimicrob Agents. 2015;45(6):635–40. 25681067 10.1016/j.ijantimicag.2014.12.022

[17] Beabout K, Hammerstrom TG, Perez AM, Magalhaes BF, Prater AG, Clements TP, Arias CA, Saxer G, Shamoo Y. The ribosomal S10 protein is a general target for decreased tigecycline susceptibility. Antimicrob Agents Chemother. 2015;59(9):5561–6. 26124155 10.1128/AAC.00547-15 PMC4538488

[18] Haeili M, Shoghi Y, Moghimi M, Ghodousi A, Omrani M, Cirillo DM. Genomic features of *in vitro* selected mutants of *Escherichia coli* with decreased susceptibility to Tigecycline. J Glob Antimicrob Resist. 2022;31:32–7. 35933109 10.1016/j.jgar.2022.07.023

[19] Band VI, Weiss DS. Heteroresistance to beta-lactam antibiotics may often be a stage in the progression to antibiotic resistance. PLoS Biol. 2021;19(7):e3001346. 34283833 10.1371/journal.pbio.3001346 PMC8351966

[20] Zhou YF, Liu P, Zhang CJ, Liao XP, Sun J, Liu YH. Colistin combined with tigecycline: a promising alternative strategy to combat *Escherichia coli* harboring Bla (NDM-) (5) and mcr-1. Front Microbiol. 2019;10: 2957. 31969868 10.3389/fmicb.2019.02957 PMC6960404

[21] Ni W, Han Y, Liu J, Wei C, Zhao J, Cui J, Wang R, Liu Y. Tigecycline treatment for Carbapenem-Resistant Enterobacteriaceae infections: a systematic review and meta-analysis. Medicine (Baltimore). 2016;95(11): e3126. 26986165 10.1097/MD.0000000000003126 PMC4839946

[22] Meletis G, Tzampaz E, Sianou E, Tzavaras I, Sofianou D. Colistin heteroresistance in carbapenemase-producing *Klebsiella pneumoniae*. J Antimicrob Chemother. 2011;66(4):946–7. 21393203 10.1093/jac/dkr007

[23] Zhang Q, Lin L, Pan Y, Chen J. Characterization of tigecycline-heteroresistant *Klebsiella pneumoniae* clinical isolates from a Chinese tertiary care teaching hospital. Front Microbiol. 2021;12:671153. 34413834 10.3389/fmicb.2021.671153 PMC8369762

[24] Schubert M, Lindgreen S, Orlando L. Adapterremoval v2: rapid adapter trimming, identification, and read merging. BMC Res Notes. 2016;9:88. 26868221 10.1186/s13104-016-1900-2 PMC4751634

[25] Bankevich A, Nurk S, Antipov D, Gurevich AA, Dvorkin M, Kulikov AS, Lesin VM, Nikolenko SI, Pham S, Prjibelski AD, et al. SPAdes: a new genome assembly algorithm and its applications to single-cell sequencing. J Comput Biol. 2012;19(5):455–77. 22506599 10.1089/cmb.2012.0021 PMC3342519

[26] Coil D, Jospin G, Darling AE. A5-miseq: an updated pipeline to assemble microbial genomes from illumina miseq data. Bioinformatics. 2015;31(4):587–9. 25338718 10.1093/bioinformatics/btu661

[27] Wick RR, Judd LM, Gorrie CL, Holt KE. Unicycler: resolving bacterial genome assemblies from short and long sequencing reads. PLoS Comput Biol. 2017;13(6):e1005595. 28594827 10.1371/journal.pcbi.1005595 PMC5481147

[28] Walker BJ, Abeel T, Shea T, Priest M, Abouelliel A, Sakthikumar S, Cuomo CA, Zeng Q, Wortman J, Young SK, et al. Pilon: an integrated tool for comprehensive microbial variant detection and genome assembly improvement. PLoS One. 2014;9(11):e112963. 25409509 10.1371/journal.pone.0112963 PMC4237348

[29] Chan PP, Lin BY, Mak AJ, Lowe TM. tRNAscan-SE 2.0: improved detection and functional classification of transfer RNA genes. Nucleic Acids Res. 2021;49(16):9077–96. 34417604 10.1093/nar/gkab688 PMC8450103

[30] Nawrocki EP, Burge SW, Bateman A, Daub J, Eberhardt RY, Eddy SR, Floden EW, Gardner PP, Jones TA, Tate J, et al. Rfam 12.0: updates to the RNA families database. Nucleic Acids Res. 2015;43(Database issue):D130–137. 25392425 10.1093/nar/gku1063 PMC4383904

[31] Akhter S, Aziz RK, Edwards RA. PhiSpy: a novel algorithm for finding prophages in bacterial genomes that combines similarity- and composition-based strategies. Nucleic Acids Res. 2012;40(16): e126. 22584627 10.1093/nar/gks406 PMC3439882

[32] Bertelli C, Laird MR, Williams KP, Simon Fraser University Research, Computing G, Lau BY, Hoad G, Winsor GL, Brinkman FSL. IslandViewer 4: expanded prediction of genomic Islands for larger-scale datasets. Nucleic Acids Res. 2017;45(W1):W30–5. 28472413 10.1093/nar/gkx343 PMC5570257

[33] Zhang J, Yu L, Fu Y, Zhao Y, Wang Y, Zhao J, Guo Y, Li C, Zhang X. Tigecycline in combination with other antibiotics against clinical isolates of carbapenem-resistant *Klebsiella pneumoniae* in vitro. Ann Palliat Med. 2019;8(5):622–31. 31735038 10.21037/apm.2019.09.11

[34] Meagher AK, Ambrose PG, Grasela TH, Ellis-Grosse EJ. Pharmacokinetic/pharmacodynamic profile for tigecycline-a new glycylcycline antimicrobial agent. Diagn Microbiol Infect Dis. 2005;52(3):165–71. 16105560 10.1016/j.diagmicrobio.2005.05.006

[35] Kulengowski B, Rutter WC, Campion JJ, Lee GC, Feola DJ, Burgess DS. Effect of increasing meropenem MIC on the killing activity of meropenem in combination with amikacin or polymyxin B against MBL- and KPC-producing *Enterobacter cloacae*. Diagn Microbiol Infect Dis. 2018;92(3):262–6. 30098852 10.1016/j.diagmicrobio.2018.06.013

[36] Yu W, Shen P, Bao Z, Zhou K, Zheng B, Ji J, Guo L, Huang C, Xiao Y. In vitro antibacterial activity of fosfomycin combined with other antimicrobials against KPC-producing *Klebsiella pneumoniae*. Int J Antimicrob Agents. 2017;50(2):237–41. 28648647 10.1016/j.ijantimicag.2017.03.011

[37] Garrison MW. Comparative antimicrobial activity of levofloxacin and ciprofloxacin against *Streptococcus pneumoniae*. J Antimicrob Chemother. 2003;52(3):503–6. 12917240 10.1093/jac/dkg380

[38] Zhao Y, Li C, Zhang J, Fu Y, Hu K, Su S, Wang Y, Li H, Zhang X. The *in vitro* activity of polymyxin B and Tigecycline alone and combination with other antibiotics against carbapenem-resistant *Enterobacter cloacae* complex isolates, including high-risk clones. Ann Transl Med. 2019;7(23):779. 32042795 10.21037/atm.2019.11.33 PMC6990000

[39] Doern CD. When does 2 plus 2 equal 5? A review of antimicrobial synergy testing. J Clin Microbiol. 2014;52(12):4124–8. 24920779 10.1128/JCM.01121-14 PMC4313275

[40] Petersen PJ, Labthavikul P, Jones CH, Bradford PA. In vitro antibacterial activities of tigecycline in combination with other antimicrobial agents determined by chequerboard and time-kill kinetic analysis. J Antimicrob Chemother. 2006;57(3):573–6. 16431863 10.1093/jac/dki477

[41] Holden ER, Webber MA. MarA, Rama, and SoxS as mediators of the stress response: survival at a cost. Front Microbiol. 2020;11:828. 32431683 10.3389/fmicb.2020.00828 PMC7216687

[42] Wang H, Dzink-Fox JL, Chen M, Levy SB. Genetic characterization of highly fluoroquinolone-resistant clinical *Escherichia coli* strains from china: role of AcrR mutations. Antimicrob Agents Chemother. 2001;45(5):1515–21. 11302820 10.1128/AAC.45.5.1515-1521.2001 PMC90498

[43] Sheng ZK, Hu F, Wang W, Guo Q, Chen Z, Xu X, Zhu D, Wang M. Mechanisms of Tigecycline resistance among *Klebsiella pneumoniae* clinical isolates. Antimicrob Agents Chemother. 2014;58(11):6982–5. 25182649 10.1128/AAC.03808-14 PMC4249433

[44] Rosenblum R, Khan E, Gonzalez G, Hasan R, Schneiders T. Genetic regulation of the RamA locus and its expression in clinical isolates of *Klebsiella pneumoniae*. Int J Antimicrob Agents. 2011;38(1):39–45. 21514798 10.1016/j.ijantimicag.2011.02.012 PMC3117140

[45] Eltai NO, Kelly B, Al-Mana HA, Ibrahim EB, Yassine HM, Al Thani A, Al Maslmani M, Lammens C, Xavier BB, Malhotra-Kumar S. Identification of *mcr*-8 in clinical isolates from Qatar and evaluation of their antimicrobial profiles. Front Microbiol. 2020;11: 1954. 32983006 10.3389/fmicb.2020.01954 PMC7476323

[46] Pitt ME, Elliott AG, Cao MD, Ganesamoorthy D, Karaiskos I, Giamarellou H, Abboud CS, Blaskovich MAT, Cooper MA, Coin LJM. Multifactorial chromosomal variants regulate polymyxin resistance in extensively drug-resistant Klebsiella pneumoniae. Microb Genom 2018, 4(3). 10.1099/mgen.0.000158 PMC5885010 29431605

[47] Yang Y, Yang Y, Chen G, Lin M, Chen Y, He R, Galvao KN, El-Gawad El-Sayed Ahmed MA, Roberts AP, Wu Y, et al. Molecular characterization of carbapenem-resistant and virulent plasmids in *Klebsiella pneumoniae* from patients with bloodstream infections in China. Emerg Microbes Infect. 2021;10(1):700–9. 33739229 10.1080/22221751.2021.1906163 PMC8023600

[48] El-Halfawy OM, Valvano MA. Antimicrobial heteroresistance: an emerging field in need of clarity. Clin Microbiol Rev. 2015;28(1):191–207. 25567227 10.1128/CMR.00058-14 PMC4284305

[49] Tian Y, Zhang Q, Wen L, Chen J. Combined effect of polymyxin B and tigecycline to overcome heteroresistance in Carbapenem-Resistant *Klebsiella pneumoniae*. Microbiol Spectr. 2021;9(2):e0015221. 34704782 10.1128/Spectrum.00152-21 PMC8549724

[50] Gil-Gil T, Berryhill BA, Manuel JA, Smith AP, McCall IC, Baquero F, Levin BR. The evolution of heteroresistance via small colony variants in *Escherichia coli* following long term exposure to bacteriostatic antibiotics. bioRxiv. 2024. 10.1038/s41467-024-52166-z PMC11391013 39261449

[51] Bernatova S, Samek O, Pilat Z, Sery M, Jezek J, Jakl P, Siler M, Krzyzanek V, Zemanek P, Hola V, et al. Following the mechanisms of bacteriostatic versus bactericidal action using raman spectroscopy. Molecules. 2013;18(11):13188–99. 24284484 10.3390/molecules181113188 PMC6270526

[52] Ma X, He Y, Yu X, Cai Y, Zeng J, Cai R, Lu Y, Chen L, Chen C, Huang B. Ceftazidime/avibactam improves the antibacterial efficacy of polymyxin B against polymyxin B heteroresistant KPC-2-producing *Klebsiella pneumoniae* and hinders emergence of resistant subpopulation *in vitro*. Front Microbiol. 2019;10:2029. 31551966 10.3389/fmicb.2019.02029 PMC6735287

[53] Band VI, Satola SW, Burd EM, Farley MM, Jacob JT, Weiss DS. Carbapenem-resistant *Klebsiella pneumoniae* exhibiting clinically undetected colistin heteroresistance leads to treatment failure in a murine model of infection. mBio. 2018. 10.1128/mBio.02448-17. 29511071 10.1128/mBio.02448-17 PMC5844991

[54] Diep JK, Sharma R, Ellis-Grosse EJ, Abboud CS, Rao GG. Evaluation of activity and emergence of resistance of polymyxin B and ZTI-01 (fosfomycin for injection) against KPC-producing *Klebsiella pneumoniae*. Antimicrob Agents Chemother. 2018. 10.1128/AAC.01815-17. 29203494 10.1128/AAC.01815-17 PMC5786778

[55] Sharma R, Patel S, Abboud C, Diep J, Ly NS, Pogue JM, Kaye KS, Li J, Rao GG. Polymyxin B in combination with meropenem against carbapenemase-producing *Klebsiella pneumoniae*: pharmacodynamics and morphological changes. Int J Antimicrob Agents. 2017;49(2):224–32. 28040408 10.1016/j.ijantimicag.2016.10.025 PMC5786158

[56] Ni W, Wei C, Zhou C, Zhao J, Liang B, Cui J, Wang R, Liu Y. Tigecycline-amikacin combination effectively suppresses the selection of resistance in clinical isolates of KPC-producing *Klebsiella pneumoniae*. Front Microbiol. 2016;7:1304. 27594855 10.3389/fmicb.2016.01304 PMC4990548

[57] Wu H, Feng H, He L, Zhang H, Xu P. In vitro activities of Tigecycline in combination with Amikacin or colistin against Carbapenem-Resistant *Acinetobacter baumannii*. Appl Biochem Biotechnol. 2021;193(12):3867–76. 34524633 10.1007/s12010-021-03664-z

[58] Demirlenk YM, Gücer LS, Uçku D, Tanrıöver C, Akyol M, Kalay Z, Barçın E, Akcan RE, Can F, Gönen M, et al. A meta-analysis for the role of aminoglycosides and tigecyclines in combined regimens against colistin- and carbapenem-resistant *Klebsiella pneumoniae* bloodstream infections. Eur J Clin Microbiol Infect Dis. 2022;41(5):761–9. 35303195 10.1007/s10096-022-04429-0

