# Supplementary material for: Heteroresistance is associated with mutations during low concentration of tigecycline therapy in multiple-resistant Klebsiella pneumoniae
Source: Ann Clin Microbiol Antimicrob. 2025 Sep 30;24:53. doi: 10.1186/s12941-025-00815-6 (PMC12482530; doi:10.1186/s12941-025-00815-6)
Supplement: Supplementary file 1 — Supplementary material 1. [file 12941_2025_815_MOESM1_ESM.docx]

Supplementary Material

**Table S1** List of primers used in experiments

| Primers | Sequence(5’-3’) | References |
| --- | --- | --- |
| Real-time PCR | | |
| *acrA* F | ATGTGACGATAAACCGGCTC | [1] |
| *acrA* R | CTGGCAGTTCGGTGGTTATT |  |
| *acrB* F | CGATAACCTGATGTACATGTCC | [2] |
| *acrB* R | CCGACAACCATCAGGAAGCT |  |
| *tolC* F | CTACAAACAGGCGGTGGTCT | [3] |
| *tolC* R | TGTTCAGCTCGTTGATCAGG |  |
| *soxS* F | GCATCACGGTACGGAACAT | [4] |
| *soxS* R | AGTCGCCAGAAAGTCAGGAT |  |
| *ramA* F | GCATCAACCGCTGCGTATT | [5] |
| *ramA* R | CGTTGCAGATGCCATTTCG |  |
| *oqxA* F | TATCGCGCCACGCTGGAACAGG | [6] |
| *oqxA* R | CGGCGCTGCTCCCACTCTTC |  |
| *oqxB* F | GATCAGGCGCAGGTTCAGGTGCA | [6] |
| *oqxB* R | AACAGATGCACCACCAGCGTCAG |  |
| *rrsE* F | TTGACGTTACCCGCAGAAGAA | [5] |
| *rrsE* R | GCTTGCACCCTCCGTATTACC |  |
| Sequencing |  |  |
| *ramR* F | CACGGTTCATATCCTGACCA | [7] |
| *ramR* R | CCRTCGACCTTAAACACGTC |  |
| *marA-marR* F | CATAGCTGAGGCTGGAGRCC | [7] |
| *marA-marR* R | TCGGCCAATTCATAATGTTG |  |
| *soxS-soxR* F | CGGAACCTCCATCAACAGATT | [7] |
| *soxS-soxR* R | GCAGGTAAGCTGGCTCTACAA |  |
| *rpsJ* F | GGATCCCAATCGTAATGGGTATGAGGAG | [8] |
| *rpsJ* R | GGATCCTAACACGGTTTGCTTCAACTT |  |
| *acrR* F | CGTAACCTCTGTAAAGTCAT | [1] |
| *acrR* R | GCTGACAAGCTCTCCGGGC |  |
| *tetA* F | GCCTTTCCTTTGGGTTCTCT | [9] |
| *tetA* R | TGTCCGACAAGTTGCATGAT |  |
| *rpsI* F | TATGCTGCCCAAAGGTCCG | [10] |
| *rpsI* R | TTCGTCCGTGTAGTCGATAC |  |

**Table S2**. General genomic features of the KH62 and KH62-R2.

| Feature ^a^ | KH62^b^ | KH62-R2^c^ |
| --- | --- | --- |
| Genome size (bp) | 1,158,136,485 bp | 1,432,665,073 bp |
| GC content (%) | 56.87 | 56.79 |
| Plasmid | 2 | 2 |
| CDs | 5,226 | 5257 |
| Gene average length (bp) | 931 | 929 |
| Number of tRNAs | 86 | 86 |
| Number of rRNAs | 25 | 25 |
| Number of rRNAs | 0 | 0 |
| Number of genomic islands | 38 | 38 |
| Number of insertion squences | 39 | 39 |
| Genes assigned to COGs | 5161 | 5159 |
| Genes assigned to KEGG | 5898 | 5884 |
| Genes assigned to PHI | 1843 | 1840 |
| Genes assigned to VFDB | 210 | 210 |
| Genes related to antibiotic resistance | 7 | 7 |
| Repeated regions (%) | 0.35 | 0.35 |
| Phage regions | 42 | 42 |
| CRISPRs | 3 | 3 |

^a^ CDs: Complete coding sequence. COGs, Cluster of Orthologous Groups of protein; KEGG, Kyoto Encyclopedia of Genes and Genomes; PHI, Pathogen-Host Interactions database; VFDB, Virulence Factors of Pathogenic Bacteria.

^b^ The accession number of amikacin-heteroresistant *K. pneumoniae* KH62 are CP170749-CP170751.

^c^ The accession number of amikacin resistant subpopulation KH62-R2 are CP171385-CP171387.

**Table S3**. General genomic features of the KH268 and KH268-R1.

| Feature ^a^ | KH268^b^ | KH268-R1^c^ |
| --- | --- | --- |
| Genome size (bp) | 1,257,009,450 bp | 1,177,868,594 bp |
| GC content (%) | 57.00 | 56.97 |
| Number of plasmid | 4 | 5 |
| CDs | 5556 | 5481 |
| Gene average length (bp) | 912 | 912 |
| Number of tRNAs | 85 | 85 |
| Number of rRNAs | 25 | 25 |
| Number of sRNAs | 0 | 0 |
| Number of genomic islands | 56 | 56 |
| Number of insertion squences | 76 | 65 |
| Genes assigned to COGs | 5355 | 5312 |
| Genes assigned to KEGG | 5902 | 5869 |
| Genes assigned to PHI | 1819 | 1817 |
| Genes assigned to VFDB | 206 | 207 |
| Genes related to antibiotic resistance | 9 | 5 |
| Repeated regions (%) | 0.64 | 0.67 |
| Phage regions | 46 | 46 |
| CRISPRs | 2 | 2 |

^a^ CDs: Complete coding sequence. COGs, Cluster of Orthologous Groups of protein; KEGG, Kyoto Encyclopedia of Genes and Genomes; PHI, Pathogen-Host Interactions database; VFDB, Virulence Factors of Pathogenic Bacteria.

^b^ The accession number of amikacin-heteroresistant *K. pneumoniae* KH268 are CP171444-CP171448.

^c^ The accession number of amikacin resistant subpopulation KH268-R1 are CP171389-CP171393.

**Figure S1.**


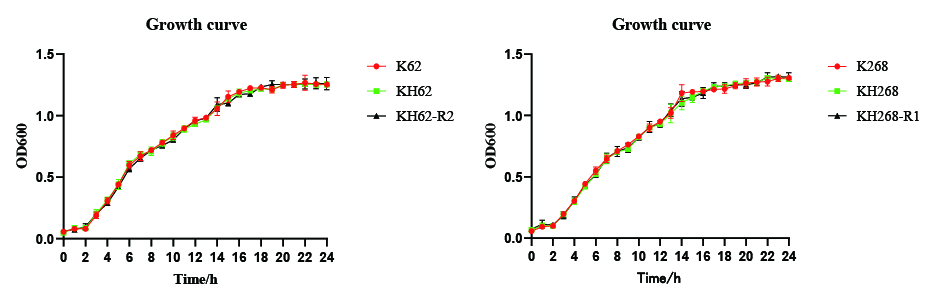


**Figure S1.** Growth curves of the isolates(K62 and K268),parental strains (KH62 and KH268) and their resistant subclones(KH62-R2 and KH268-R1).

**Figure S2.**


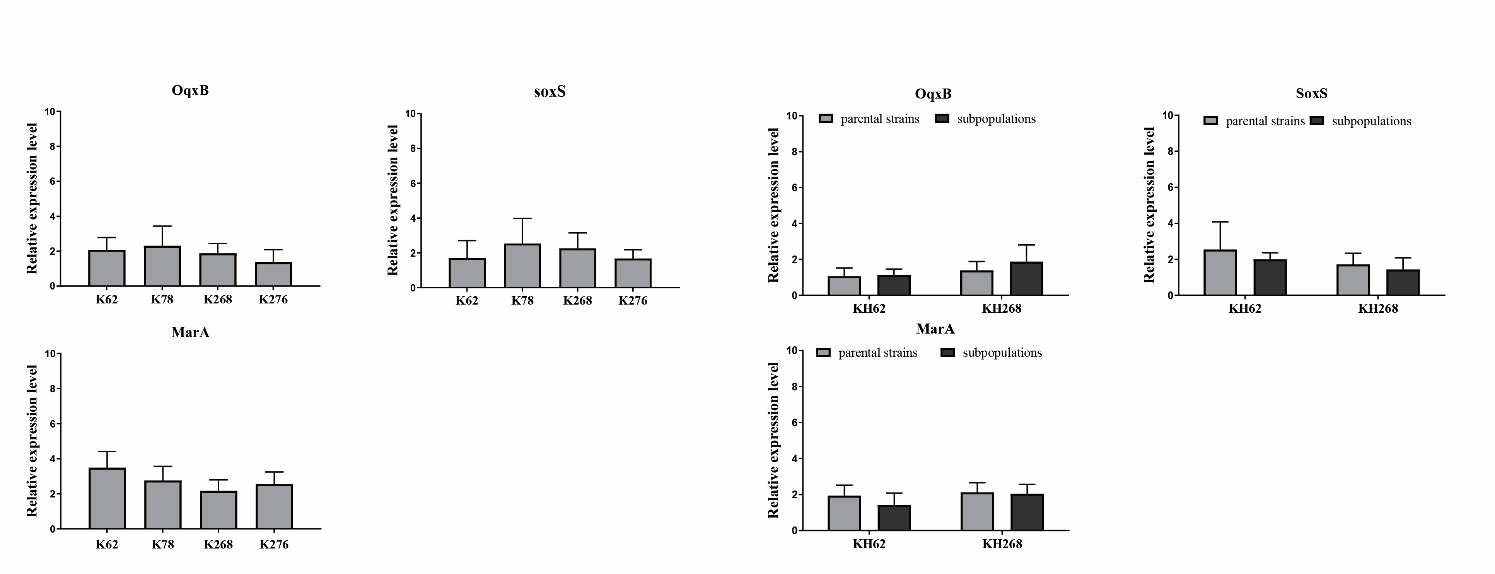


**Figure S2.** Expression of the efflux pump gene OqxB and regulator SoxS and MarA between clinical isolates, their evaluated TGCHR-Kp and resistant subpopulations.

**Figure S3.**


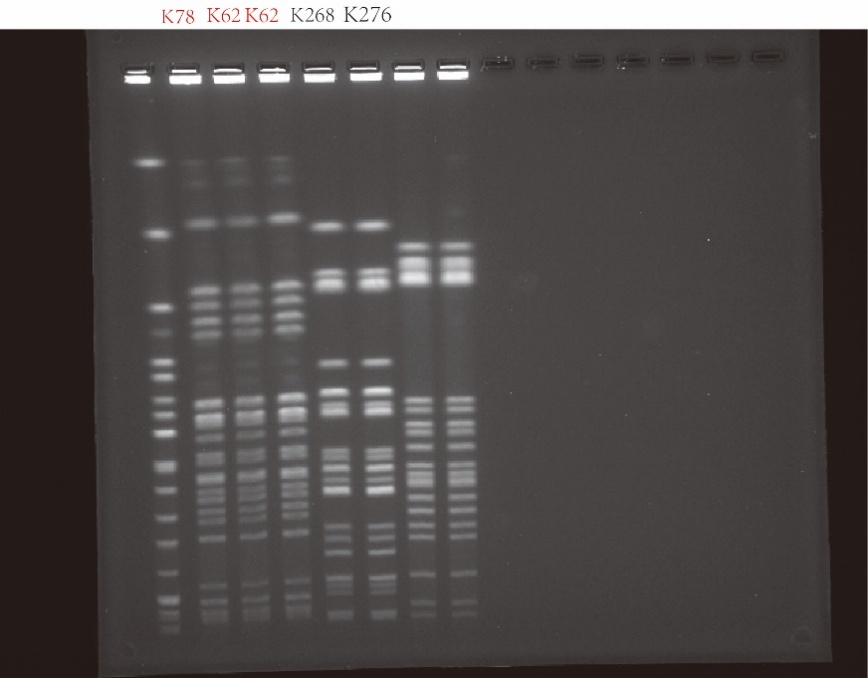


**Figure S3.** Full uncropped PFGE image(s). The homology between clinical isolates K78, K62 and K268, K276 were analyzed by PFGE.

**Reference**

1. Kallman O, Motakefi A, Wretlind B, Kalin M, Olsson-Liljequist B, Giske CG: **Cefuroxime non-susceptibility in multidrug-resistant Klebsiella pneumoniae overexpressing ramA and acrA and expressing ompK35 at reduced levels**. *J Antimicrob Chemother* 2008, **62**(5):986-990.

2. Doumith M, Ellington MJ, Livermore DM, Woodford N: **Molecular mechanisms disrupting porin expression in ertapenem-resistant Klebsiella and Enterobacter spp. clinical isolates from the UK**. *J Antimicrob Chemother* 2009, **63**(4):659-667.

3. Roy S, Datta S, Viswanathan R, Singh AK, Basu S: **Tigecycline susceptibility in Klebsiella pneumoniae and Escherichia coli causing neonatal septicaemia (2007-10) and role of an efflux pump in tigecycline non-susceptibility**. *J Antimicrob Chemother* 2013, **68**(5):1036-1042.

4. Bratu S, Landman D, George A, Salvani J, Quale J: **Correlation of the expression of acrB and the regulatory genes marA, soxS and ramA with antimicrobial resistance in clinical isolates of Klebsiella pneumoniae endemic to New York City**. *J Antimicrob Chemother* 2009, **64**(2):278-283.

5. Ruzin A, Visalli MA, Keeney D, Bradford PA: **Influence of transcriptional activator RamA on expression of multidrug efflux pump AcrAB and tigecycline susceptibility in Klebsiella pneumoniae**. *Antimicrobial agents and chemotherapy* 2005, **49**(3):1017-1022.

6. Liu H, Jia X, Zou H, Sun S, Li S, Wang Y, Xia Y: **Detection and characterization of tigecycline heteroresistance in E. cloacae: clinical and microbiological findings**. *Emerg Microbes Infect* 2019, **8**(1):564-574.

7. Bialek-Davenet S, Marcon E, Leflon-Guibout V, Lavigne JP, Bert F, Moreau R, Nicolas-Chanoine MH: **In vitro selection of ramR and soxR mutants overexpressing efflux systems by fluoroquinolones as well as cefoxitin in Klebsiella pneumoniae**. *Antimicrob Agents Chemother* 2011, **55**(6):2795-2802.

8. Fang L, Chen Q, Shi K, Li X, Shi Q, He F, Zhou J, Yu Y, Hua X: **Step-Wise Increase in Tigecycline Resistance in Klebsiella pneumoniae Associated with Mutations in ramR, lon and rpsJ**. *PLoS One* 2016, **11**(10):e0165019.

9. Akiyama T, Presedo J, Khan AA: **The tetA gene decreases tigecycline sensitivity of Salmonella enterica isolates**. *International journal of antimicrobial agents* 2013, **42**(2):133-140.

10. Duffin PM, Seifert HS: **ksgA mutations confer resistance to kasugamycin in Neisseria gonorrhoeae**. *Int J Antimicrob Agents* 2009, **33**(4):321-327.
